# A dangerously underrated entity? Non-specific complaints at emergency department presentation are associated with utilisation of less diagnostic resources

**DOI:** 10.1186/s12873-021-00531-2

**Published:** 2021-11-10

**Authors:** Tanja Birrenbach, Andrea Geissbühler, Aristomenis K. Exadaktylos, Wolf E. Hautz, Thomas C. Sauter, Martin Müller

**Affiliations:** 1 Department of Emergency Medicine, Inselspital, University Hospital, University of Bern, Bern, Switzerland; 2 Institute of Health Economics and Clinical Epidemiology, University Hospital of Cologne, Cologne, Germany

**Keywords:** Non-specific complaints, Resource utilisation, Emergency department, Adult

## Abstract

**Background:**

Patients presenting with non-specific complaints (NSC), such as generalised weakness, or feeling unwell, constitute about 20% of emergency care consultations. In contrast to patients presenting with specific symptoms, these patients experience more hospitalisations, longer stays in hospital and even higher mortality. However, little is known about the actual resources spent on patients with NSC in the emergency department (ED).

**Methods:**

We have conducted a retrospective analysis from January 1st, 2013 until December 31st, 2017 in a Swiss tertiary care ED to assess the impact of NSC on the utilisation of diagnostic resources in adult patients with highlyurgent or urgent medical complaints.

**Results:**

We randomly selected 1500 medical consultations from our electronic health record database: The majority of patients (*n* = 1310, 87.3%) presented with a specific complaint; *n* = 190 (12.7%) with a NSC. Univariate analysis showed no significant difference in the utilisation of total diagnostic resources in the ED [specific complaints: 844 (577–1313) vs. NSC: 778 (551–1183) tax points, *p* = 0.092, median (interquartile range)]. A backward selection logistic regression model was adjusted for the identified covariates (age, diabetes, cerebrovascular and liver disease, malignancy, past myocardial infarction, antihypertensive, antithrombotic or antidiabetic medication, night or weekend admission and triage category). This identified a significant association of NSC with lower utilisation of ED diagnostic resources [geometric mean ratio (GMR) 0.91, 95% CI: 0.84–0.99, *p* = 0.042].

**Conclusions:**

Non-specific complaints (NSC) are a frequent reason for emergency medicine consultations and are associated with lower utilisation of diagnostic resources during ED diagnostic testing than with specific complaints.

**Supplementary Information:**

The online version contains supplementary material available at 10.1186/s12873-021-00531-2.

## Background

Non-specific complaints (NSC), such as generalised weakness and fatigue or feeling unwell, make up a large group of complaints in emergency care [1–3]. These “common unknown unknowns” [4] encompass a large number of possible, even life-threatening diagnoses [1, 5–9]. These patients often present a diagnostic challenge to the attending physician, as there is no universal definition and no specific management algorithms. It is not surprising that this leads to a high rate of misdiagnosis [10], with serious or even fatal consequences for the patient. We recently demonstrated that mortality is higher in hospitalised patients presenting with NSC to our emergency department (ED) [11] than for patients with specific complaints; this is consistent with reports from other countries [1, 3, 6, 7, 12–14]. This statement can also be confirmed when looking at the entire ED population (all patients admitted and discharged) [7, 12, 14, 15].

In the US, ED visits increased by 44% from 1996 to 2010 [16], and similar trends have been reported in other developed countries [17], as well as in Switzerland [18, 19], and this contributes to the burden of rising health care costs. EDs serve as important points of entry to the health care system and are a pivotal source of medical care. As expected, utilisation of ED diagnostic resources (and the corresponding costs) increases with both age [20] and symptom severity (triage level) [21, 22].

Very little is known about the utilisation of diagnostic resources by patients presenting with NSC. In their cohort of elderly patients presenting to the ED with weakness and fatigue, Bhalla et al. found a significant increase in the number of diagnostic tests, and procedures [2]. However, these results were not confirmed in another cohort of elderly patients presenting to the ED with NSC [7]. A recent systematic review on NSC in the ED found that the data on resources required were insufficient for further analysis [14].

As the number of ED consultations and overall health costs are both increasing and in view of the growing awareness of the important but largely unknown entity of patients with NSC, we have investigated the impact of NSC on the utilisation of diagnostic resources in the ED; to our knowledge, this has never been comprehensively studied. Our working hypothesis was that resource utilisation was greater in adult patients presenting to the ED with a medical NSC who were triaged as highlyurgent or urgent (all-comers).

## Methods

### Study design, setting, and ethical approval

This study is a retrospective analysis at the ED of the Bern University Hospital in Bern, Switzerland. Our ED is a tertiary care centre, caring for a patient population of around 2 million and treating over 50,000 adult patients each year with an interdisciplinary team [23]. All patients are triaged by registered nurses using the Swiss triage scale that encompasses four levels (life-threatening, highlyurgent, urgent, non-urgent) [24]. Every Swiss inhabitant is obliged to obtain healthcare insurance that - in its base tariff - covers the costs of healthcare and medication. Private complementary medical insurance is available to pay for services not covered by mandatory health insurance (e.g. complementary medicine, acupuncture), in order to ensure free choice of hospitals or doctors and preferred hospital accommodation. However, this has no influence on emergency examinations, since in Switzerland these are reimbursed as follows: In the ED, all procedures are coded by the person who performed the procedure - using a procedural code of the *Tarmed Suisse catalogue* (TARMED Suisse. TARMED 01.08.0000). For each procedural code, a number measured in *tax points* is assigned, that reflects the effort of the procedure, independently of medical insurance status. There is no flat rate per case or DRG system (diagnosis related groups) for emergency treatments. The tax point is a hospital-specific, medical currency (1 tax point is roughly 1 Swiss Franc) and may depend on the individual hospital.

This study was classified as a quality evaluation study by the local institutional review board (KEK-2018-00198) and the need for informed consent was waived.

### Inclusion/exclusion

**Fig. 1:**
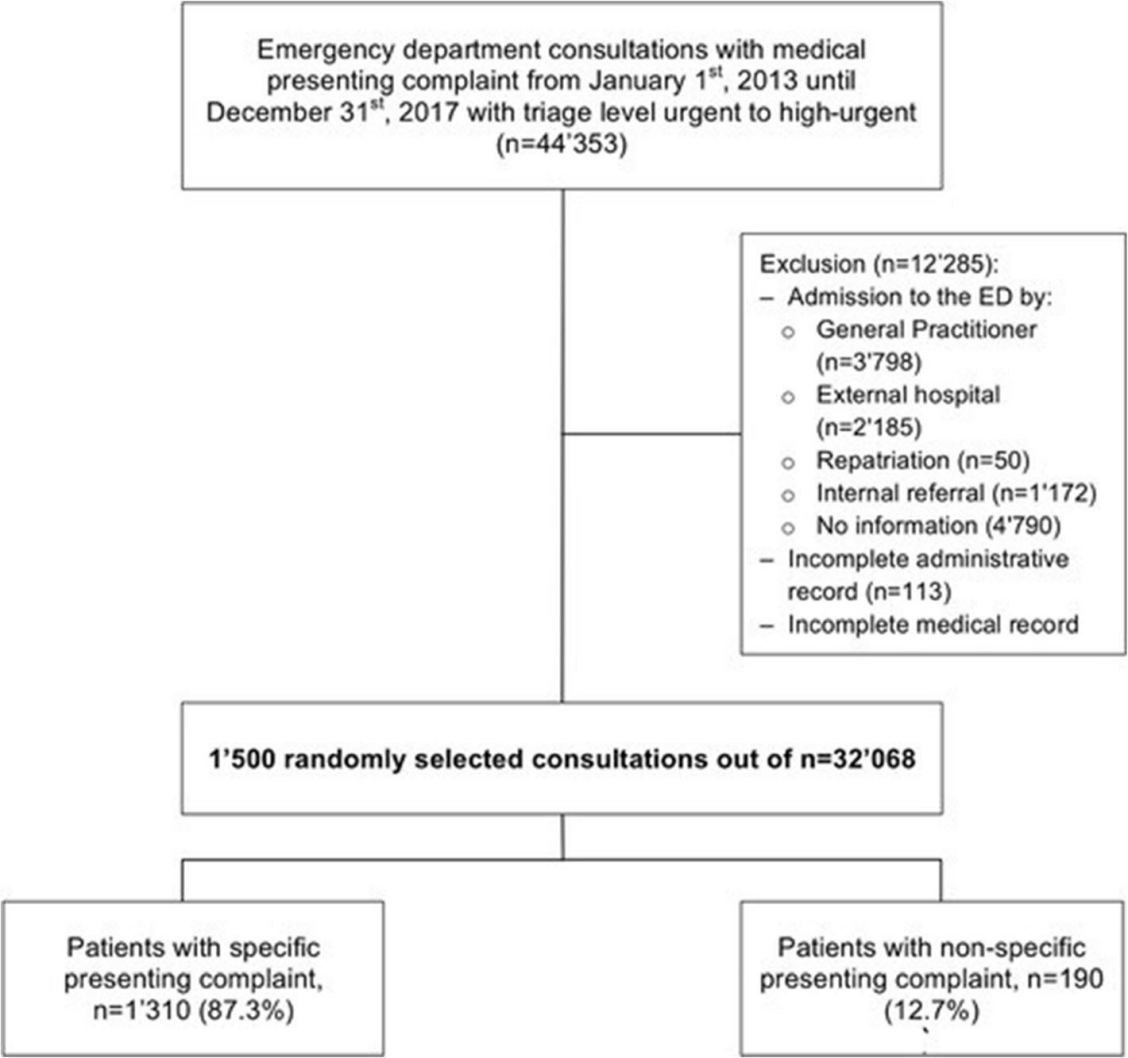
Flowchart

We randomly chose 1500 patients encounters from all adult patients (> 16 years of age) presenting to the ED between January 1st, 2013 and December 31st, 2017 triaged as urgent to highly-urgent, with a medical presenting complaint (Fig. 1. Flowchart). Life-threatening emergencies (around 8% of our patients in the ED) were excluded, as these are generally cared for within one of the three resuscitation bays and usually consume a lot of resources in a short period of time, as they do not stay long until being referred for further treatment (i.e. ICU, operation theatre). Non-urgent acute patients are assessed as being in a stable condition of health that does not actually require emergency medical therapy, and were therefore excluded (i.e. follow-up, concern related to a non-urgent health problem, medical certificate that is required for a specific administrative purpose, loss of a medical prescription).

Patients were sampled from all patients with complete medical and administrative records, and no previous medical contact for the current admission to the ED (previously seen by general practitioner, internal (i.e. specialist clinic) or external (i.e. external hospital, external specialist) referral, repatriation from a hospital in another country).

### Data collection & extraction

*Sociodemographic* (age, sex, Swiss nationality, private complementary medical insurance and *medical* (important comorbidities and drug intake) *patient characteristics*, *encounter characteristics* (time and day of presentation (night-time admission to ED 7 pm-7 am; effective weekend admission to ED 7 pm Friday to 7 am Monday), transport by emergency medical service (EMS), Swiss triage scale, and *patient outcome data* (length of stay in the ED and hospital, intensive care unit (ICU) admission, in-hospital mortality, ED return visits for 30 and 365 days, respectively), as well as *study outcome data* (physician and nursing, material and medication, laboratory, and radiological resources) were collected from the hospital’s electronic patient documentation systems (E-Care, ED 2.1.3.0, Turnhout Belgium and i-pdos_Prod_ODA 7.10.1.2).

Important comorbidities i.e. COPD, diabetes, liver disease, dementia, malignancy, cerebrovascular disease, peripheral artery disease, past myocardial infarction, and chronic kidney disease in the diagnosis list and specific drug intake were determined through a previously validated full-text parser of the ED medical report (see S1 Table). The Anatomical Therapeutic Chemical (ATC) classification system was used to categorise medication intake. The following groups were used: antidiabetics (ATC code A10), antithrombotics (B01), antihypertensives (C02, C04-C09), diuretics (C03), opioids (N02A), antiepileptics (N03), and psycholeptics (N05) [25].

### Exposure: non-specific complaints

Nemec et al. defined NSC as an entity of complaints that is not part of the set of specific complaints or signs for which evidence-based management protocols for the ED physician exist (e.g. “generalised weakness”, “decreased general condition”, “feeling exhausted”, “dizziness”) [13]. As they also excluded trauma patients, we focussed our investigations on patients presenting with medical complaints. Other investigators have applied the same definition using this “rule-out” or “absence of specific symptoms” [5, 11]. The presenting complaint collected from the hospital’s electronic patient documentation system was classified as either specific or non-specific according to the proposed scheme by two authors independently (TB, AG). In case of discrepancies between the two reviewers, a third reviewer (TCS) was involved.

### Primary and secondary outcomes

The primary outcome was the total utilisation of ED diagnostic resources, defined as the sum of all physician resources (physician patient = consultation time; physician admin = physician administrative work; physician medical record; physician other = other physician services, e.g. arterial puncture; counselling = specific medical counselling, e.g. smoking cessation), as well as nursing, material (e.g. wound dressing, intravenous catheter, but also including medication costs), laboratory, and radiological resources. In the ED, all procedures are coded by the person who performed the procedure - using a procedural code of the *Tarmed Suisse catalogue* (TARMED Suisse. TARMED 01.08.0000). For each procedural code, a number measured in *tax points* is assigned, that reflects the effort of the procedure. The tax point is a hospital-specific, medical currency (1 tax point is roughly 1 Swiss Franc) and may depend on the individual hospital.

Secondary outcomes were hospitalisation, length of stay in the ED and hospital, ICU admission, in-hospital mortality, as well as ED return visits within 30 and 365 days.

### Justification of sample size

The geometric mean of a distribution can be obtained by taking the exponentiated mean of the ln-transformed values. On the assumption that the mean of the ln-transformed total ED utilisation of diagnostic resources was about 6.55 (geometric mean of 700 tax points) with a standard deviation (SD) of 0.65, as based on a random sample of 100 consultations and a ratio between NSC- and non-NSC patients of 0.1, a sample size of 1500 patients is sufficient to show a difference of more than 20% in the geometric mean ratio (0.8–1.2) of the ED utilisation of diagnostic resources.

### Statistical analysis

Data was analysed in Stata® 13.1 (StataCorp, The College Station, Texas, USA).

Baseline characteristics are presented as numbers and percentages or medians and interquartile ranges (IQR), using descriptive statistics as appropriate.

To assess the interrater agreement, we used Gwet’s AC - the test parameter of choice for the case of two raters and low prevalence data [26]. Gwet’s AC > 0.8 was considered to be a “very good” agreement [27].

Groups of patients with specific or NSC were compared with respect to presentation, ED and hospital outcome, as well as utilisation of ED resources, using Chi-square and Wilcoxon rank sum tests as applicable. Logistic regressions were performed to reveal associations with a binary outcome - i.e. NSC; the effect sizes are presented as odds ratios (OR) and 95% confidence intervals (CI).

As the primary outcome, total utilisation of ED diagnostic resources, was not normally distributed, the variable was ln-transformed before analysis. To reduce the risk of confounding, variables that showed at least evidence for a weak association (*p* < 0.2) – both with the primary outcome (utilisation of ED diagnostic resources) and exposure (NSC) – were considered as potential confounders [28, 29]. A stepwise backward linear regression analysis including all potential confounders was used to control the association between NSC and utilisation of ED diagnostic resources. The effect sizes of the linear regression are presented as geometrical mean ratios (GMR) and 95% confidence intervals (CI), as the exponentiated coefficients of a linear regression analysis with ln-transformed outcome corresponds to the GMR of the outcome without ln-transformation. The likelihood-ratio test was used to test if the final model improved adding an interaction term between i) NSC and age, ii) NSC and sex, iii) NSC and high triage category, and iv) triage category and age*.*

A *p*-value < 0.05 was considered significant.

## Results

During the study period, 44,353 patients presented with a medical (as opposed to e.g. a surgical) problem and with triage level urgent to highlyurgent (Fig. 1. Flowchart). Of these patients, 12,285 were excluded due to incomplete medical (*n* = 177) or administrative records (documented resources < 10 tax points, *n* = 113), or a previous medical contact (*n* = 11,995). Of the remaining 32,068 consultations, 1500 were randomly selected. The majority of these patients (*n* = 1310, 87.3%) presented with a specific complaint, and 190 (12.7%) with an NSC.

The interrater agreement with regard to complaint specificity was very good (Gwet’s AC: 0.87, 95% CI: 0.85, 0.89).

### Characteristics of patients with specific vs. non-specific complaints

The consultation characteristics of patients with specific vs. non-specific complaints are detailed in Tables 1 and 2. Among the sociodemographic characteristics, age (per year increase) (OR 1.01, 95% CI: 1.00–1.02, *p* = 0.040), male sex (OR 1.53, 95% CI: 1.12–2.09, *p* = 0.007), and private insurance (OR 1.78, 95% CI: 1.21–2.61, *p* = 0.003) demonstrated higher odds for presenting with a NSC. In the context of multimorbidity, only the presence of diabetes and antidiabetic medication had higher odds for presenting with a NSC (OR 2.07, 95% CI: 1.36–3.15, *p* = 0.001 and OR 1.94, 95% CI: 1.20–3.13, *p* = 0.007, respectively). Weekend admissions were associated with lower odds for NSC (OR 0.65, 95% CI: 0.47–0.91, *p* = 0.011). The highly urgent triage category was significantly associated with lower odds for presenting with a NSC (OR 0.50, 95% CI: 0.36–0.70, *p* < 0.001).

**Table 1:** Consultation characteristics of patients with specific vs. non-specific complaints

	Specific (*n* = 1310)		NSC (*n* = 190)		Total (*n* = 1500)		p
**Sociodemographic characteristics**							
Age, [med (IQR)]	50.0	(33–67)	55.5	(39–68)	51.0	(34–67)	0.027
Sex, [n (%)]							
Female	640	(48.9)	73	(38.4)	713	(47.5)	
Male	670	(51.1)	117	(61.6)	787	(52.5)	0.007
Swiss nationality, [n (%)]	927	(70.8)	142	(74.7)	1069	(71.3)	0.258
Private insurance, [n (%)]	171	(13.1)	40	(21.1)	1069	(71.3)	0.003
**Transport by EMS**	373	(28.5)	46	(24.2)	419	(27.9)	0.221
**Time and day of admission to ED**							
Saturday or Sunday admission, [n (%)]	431	(32.9)	51	(26.8)	482	(32.1)	0.095
Night-time admissions, [n (%)]	593	(45.3)	74	(38.9)	667	(44.5)	0.101
Effective weekends^a^, [n (%)]	519	(39.6)	57	(30.0)	576	(38.4)	0.011
Effective days off^b^, [n (%)]	443	(33.8)	53	(27.9)	496	(33.1)	0.105
**Comorbidity**							
COPD, [n (%)]	75	(5.7)	9	(4.7)	84	(5.6)	0.580
Diabetes, [n (%)]	153	(11.7)	38	(20.0)	191	(12.7)	0.001
Liver disease, [n (%)]	89	(6.8)	16	(8.4)	105	(7.0)	0.411
Dementia, [n (%)]	27	(2.1)	6	(3.2)	33	(2.2)	0.335
Past myocardial infarction, [n (%)]	181	(13.8)	20	(10.5)	201	(13.4)	0.213
Malignancy, [n (%)]	204	(15.6)	35	(18.4)	239	(15.9)	0.316
Peripheral artery disease, [n (%)]	42	(3.2)	4	(2.1)	46	(3.1)	0.411
Chronic kidney disease, [n (%)]	51	(3.9)	9	(4.7)	60	(4.0)	0.579
Cerebrovascular disease, [n (%)]	98	(7.5)	16	(8.4)	114	(7.6)	0.648
**Drug intake**							
On any antidiabetic, [n (%)]	104	(7.9)	27	(14.2)	131	(8.7)	0.004
On any antithrombotic, [n (%)]	372	(28.4)	59	(31.1)	431	(28.7)	0.450
On any antihypertensive, [n (%)]	411	(31.4)	69	(36.3)	480	(32.0)	0.172
On any diuretic, [n (%)]	178	(13.6)	25	(13.2)	203	(13.5)	0.871
On any antibiotic, [n (%)]	188	(14.4)	15	(7.9)	203	(13.5)	0.015
On any antiepileptic, [n (%)]	108	(8.2)	14	(7.4)	122	(8.1)	0.680
On any opioids, [n (%)]	97	(7.4)	15	(7.9)	112	(7.5)	0.810
On any psycholeptic, [n (%)]	178	(13.6)	26	(13.7)	204	(13.6)	0.971
**Consultation characteristic**							
Triage category, [med (iqr)]	3.0	(2, 3)	3.0	(2, 3)	3.0	(2, 3)	< 0.001
Highly-urgent	588	(44.9)	55	(28.9)	643	(42.9)	
Urgent	722	(55.1)	135	(71.1)	857	(57.1)	< 0.001
Resuscitation bay, [n (%)]	17	(1.3)	2	(1.1)	19	(1.3)	0.778
**Outcomes**							
Hospitalisation, [n (%)]	516	(39.4)	72	(37.9)	588	(39.2)	0.693
30 d revisits, [n (%)]	175	(13.4)	22	(11.6)	197	(13.1)	0.497
365 d revisits, [n (%)]	476	(36.3)	73	(38.4)	549	(36.6)	0.577
LOS hospital days, [med (iqr)]	0.3	(0.2–3.4)	0.3	(0.2–3.3)	0.3	(0.2–3.4)	0.940
LOS ED hours, [med (iqr)]	4.7	(3.4–6.5)	4.9	(3.3–6.8)	4.7	(3.3–6.6)	0.871
ICU admission, [n (%)]	108	(8.2)	13	(6.8)	121	(8.1)	0.507
In-hospital death, [n (%)]	21	(1.6)	5	(2.6)	26	(1.7)	0.310

*Abbreviations*: *COPD* chronic obstructive pulmonary disease, *ED* emergency department, *EMS* emergency medical services, *ICU* intensive care unit, *IQR* interquartile range, *LOS* length of stay, *med* median, *NSC* non-specific complaint

^a^Effective weekends: admission to ED 7 pm Friday to 7 am Monday

^b^Effective days off: effective weekends and all public holidays

**Table 2:** Associations with NSC status

	OR	95% CI	p
**Sociodemographic characteristics**			
Age, per year	1.01	(1.00–1.02)	0.040
Sex			
Female	1.00	(base)	
Male	1.53	(1.12–2.09)	0.007
Swiss nationality	1.22	(0.86–1.73)	0.259
Private insurance	1.78	(1.21–2.61)	0.003
**Transport by EMS**	0.80	(0.56–1.14)	0.221
**Time and day of admission to ED**			
Saturday or Sunday	0.75	(0.53–1.05)	0.096
Night-time admission	0.77	(0.57–1.05)	0.102
Effective weekends^a^	0.65	(0.47–0.91)	0.011
Effective days off^b^	0.76	(0.54–1.06)	0.106
**Comorbidity**			
COPD	0.69	(0.33–1.44)	0.326
Diabetes	2.07	(1.36–3.15)	0.001
Dementia	1.56	(0.61–3.95)	0.352
Past myocardial infarction	0.67	(0.40–1.11)	0.121
Malignancy	1.14	(0.76–1.72)	0.521
Peripheral artery disease	0.55	(0.19–1.62)	0.279
Chronic kidney disease	1.16	(0.54–2.46)	0.708
Cerebrovascular disease	1.14	(0.64–2.02)	0.655
**Drug intake**			
On any antidiabetic	1.94	(1.20–3.13)	0.007
On any antithrombotic	1.02	(0.69–1.53)	0.905
On any antihypertensive	1.19	(0.80–1.78)	0.397
On any diuretic	0.81	(0.49–1.33)	0.400
On any opioids	0.84	(0.46–1.53)	0.561
On any antiepileptic	1.04	(0.58–1.88)	0.893
On any psycholeptic	1.01	(0.64–1.59)	0.981
**Consultation characteristic**			
Triage category			
Urgent	1.00	(base)	
Highly-urgent	0.50	(0.36–0.70)	< 0.001
**Outcomes**			
Hospitalisation	0.94	(0.69–1.28)	0.693
30 days revisits	0.85	(0.53–1.36)	0.498
365 days revisits	1.09	(0.80–1.50)	0.577
LOS hospital days	1.01	(0.99–1.03)	0.564
LOS ED hours	0.99	(0.95–1.04)	0.777
ICU admission	0.82	(0.45–1.48)	0.508
In-hospital death	1.66	(0.62–4.45)	0.315

*Abbreviations*: *CI* confidence interval, *COPD* chronic obstructive pulmonary disease, *ED* emergency department, *EMS* emergency medical services, *ICU* intensive care unit, *LOS* length of stay, *NSC* non-specific complaint, *OR* odds ratio

^a^Effective weekends: admission to ED 7 pm Friday to 7 am Monday

^b^Effective days off: effective weekends and all public holidays

For parameters of patient outcome, no significant difference was detected. If the analysis was restricted to hospitalised patients (*n* = 588), the patients presenting with an NSC (*n* = 72) had a significantly (*p* = 0.039) longer hospital stay (median 5.8 days, IQR: 2.9–9.6) than those presenting with a specific complaint (*n* = 516) (median 4.3 days, IQR: 2.1–8.0).

### Utilisation of diagnostic resources

**Fig. 2:**
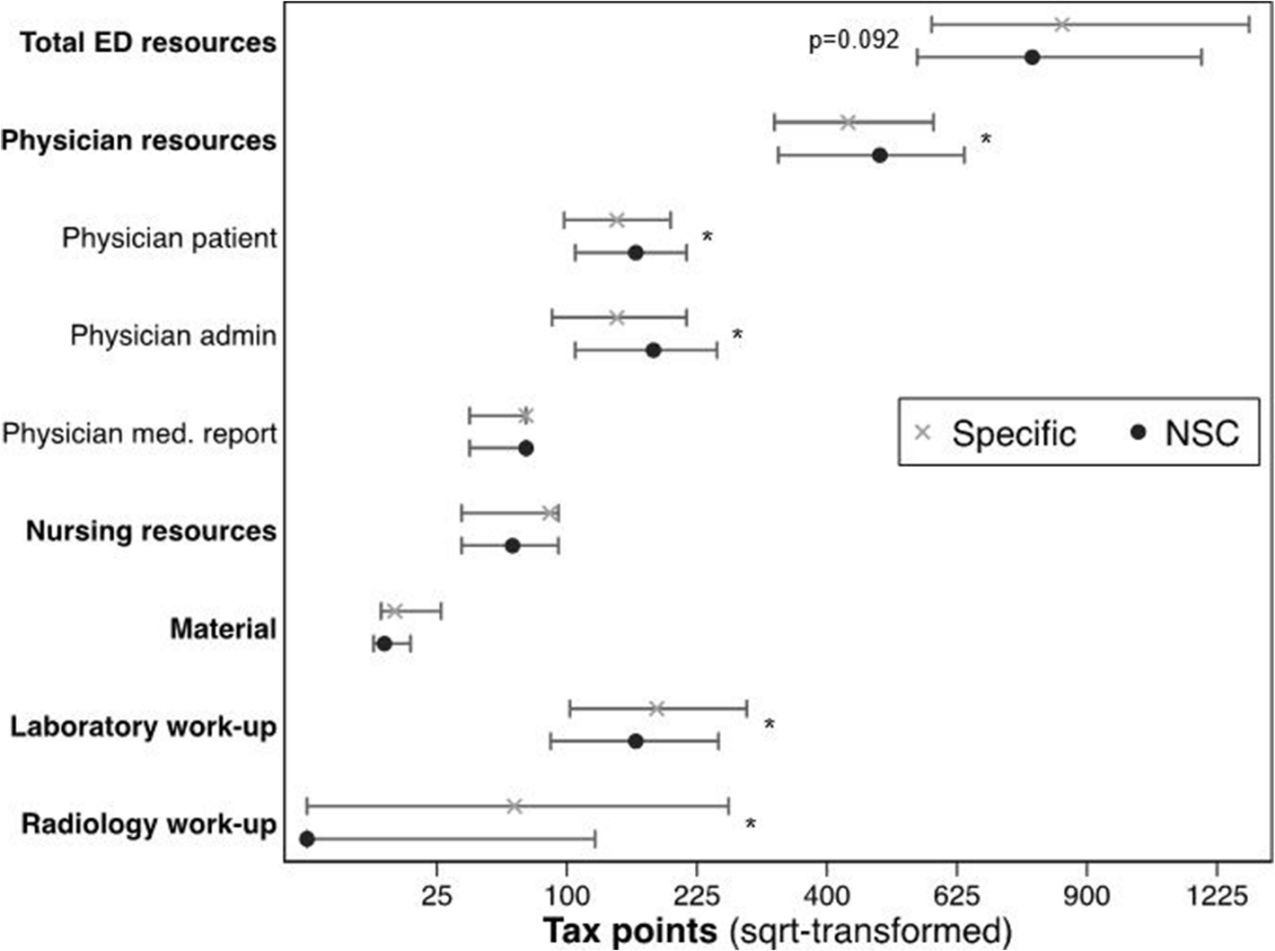
Resource utilisation measured in tax points in the ED according to NSC status. The group medians with the accompanying interquartile range are shown.* significant (*p* < 0.05) group difference

In univariate analysis, no significant difference in the total utilisation of diagnostic resources in the ED was found [specific: 844 (577–1313) vs. NSC: 778 (551–1183) tax points, *p* = 0.092, median (IQR)]. For the different resource categories, patients presenting with NSC utilised significantly more physician resources than patients presenting with a specific complaint [specific: 433 (323–581) vs. NSC: 485 (329–639) tax points, *p* = 0.048, median (IQR)]. Furthermore, utilisation of material, laboratory and radiology resources was found to be significantly less in patients presenting with a NSC (Fig. 2).

The associations of the various confounders with the total consumption of diagnostic resources are detailed in Table 3.

**Table 3:** Associations of various confounders with utilisation of ED diagnostic resources

	GMR	95% CI	p
**Sociodemographic characteristics**			
Age, per year	1.01	(1.01–1.01)	< 0.001
Sex, male	1.04	(0.98–1.11)	0.205
Swiss nationality	1.14	(1.07–1.23)	< 0.001
Private insurance	1.17	(1.07–1.28)	0.001
**Transport by EMS**	1.34	(1.25–1.43)	< 0.001
**Time and day of admission to ED**			
Saturday or Sunday admission	0.97	(0.91–1.04)	0.395
Night-time admissions	0.78	(0.74–0.83)	< 0.001
Effective weekends^a^	0.93	(0.87–0.99)	0.023
Effective days off^b^	0.98	(0.92–1.05)	0.580
**Comorbidity**			
COPD	1.17	(1.02–1.34)	0.026
Diabetes	1.14	(1.04–1.25)	0.006
Liver disease	1.16	(1.03–1.31)	0.015
Dementia	1.51	(1.22–1.87)	< 0.001
Past myocardial infarction	1.18	(1.07–1.29)	< 0.001
Malignancy	1.33	(1.22–1.44)	< 0.001
Peripheral artery disease	1.18	(0.99–1.42)	0.068
Chronic kidney disease	1.21	(1.03–1.41)	0.021
Cerebrovascular disease	1.38	(1.23–1.55)	< 0.001
**Drug intake**			
On any antidiabetic	1.18	(1.06–1.32)	0.003
On any antithrombotic	1.35	(1.27–1.45)	< 0.001
On any antihypertensive	1.31	(1.22–1.4)	< 0.001
On any diuretic	1.33	(1.22–1.46)	< 0.001
On any antiepileptic	1.04	(0.93–1.17)	0.497
On any psycholeptic	1.15	(1.02–1.29)	0.025
**Consultation characteristic**			
Triage			
Urgent	1	(base)	
Highly-urgent	1.31	(1. 22–1.39)	< 0.001
**Outcomes**			
Hospitalisation	1.58	(1.48–1.67)	< 0.001
30 d revisits	0.97	(0.88–1.06)	0.474
365 d revisits	1.01	(0.95–1.08)	0.752
LOS hospital days	1.02	(1.01–1.02)	< 0.001
LOS ED hours	1.1	(1.09–1.11)	< 0.001
ICU admission	1.55	(1.39–1.74)	< 0.001
In-hospital death	1.46	(1.15–1.85)	0.002

*Abbreviations*: *CI* confidence interval, *COPD* chronic obstructive pulmonary disease, *ED* emergency department, *EMS* emergency medical services, *GMR* geometric mean ratio, *ICU* intensive care unit, *LOS* length of stay

^a^Effective weekends: admission to ED 7 pm Friday to 7 am Monday

^b^Effective days off: effective weekends and all public holidays

A backward selection logistic regression model including relevant confounders (variables that showed at least a weak association (*p* < 0.2) – both with the primary outcome (utilisation of ED diagnostic resources) and exposure (NSC), i.e. age, private insurance, triage category, night or weekend admission and on any antidiabetic) showed that NSC was significantly associated with lower utilisation of ED diagnostic resources [geometric mean ratio (GMR) 0.91, 95% CI: 0.83–1.00, *p* = 0.040], corresponding to 9% less utilisation of total ED diagnostic resources (Table 4).

**Table 4:** Backward selection model (*p* < 0.2) including all potential predictor variables (predictors with *p* < 0.05 are highlighted in bold)

ED diagnostic resource utilisation	GMR	95% CI	p
**NSC**	0.91	(0.83–1.00)	0.040
**Age, per year**	1.01	(1.01–1.01)	< 0.001
Private insurance	1.06	(0.97–1.15)	0.197
On any antidiabetic	1.07	(0.97–1.19)	0.189
**Night admission**	0.83	(0.78–0.88)	< 0.001
Effective weekends	0.95	(0.89–1.00)	0.070
**Triage**			
Urgent (base)	1.00		
Highly-urgent	1.25	(1.17–1.32)	< 0.001

*Abbreviations*: *ED* emergency department, *CI* confidence interval, *GMR* geometric mean ration, *NSC* non-specific complaint

None of the added interaction terms (NSC and age, ii) NSC and sex, iii) NSC and high triage category, and iv) triage category and age) improved the final model significantly (*p* > 0.1 in the likelihood-ratio test for all scenarios).

## Discussion

Although NSC are among the most frequent complaints in emergency medicine [1, 2], little is known is about this particular patient group. Using a backward selection model adjusted for relevant confounders (age, private insurance, night or weekend admission, triage category, on antidiabetic medication), we found that NSC were significantly associated with lower utilisation of ED diagnostic resources in comparison to patients presenting with a specific medical complaint. To our knowledge, this is the first study to comprehensively assess the association between NSC and utilisation of diagnostic resources in the ED.

Given the vague presentation of NSC, the broad spectrum of underlying diagnoses, and the lack of diagnostic algorithms, we hypothesised that resource utilisation would be higher in this category of patients. However, in an uncorrected comparison, total diagnostic resources utilised in the ED were not different and even significantly lower in the NSC group after adjusting for relevant confounders. A detailed comparison of the apportionment of resource utilisation showed that the total amount of physician resources spent (mainly in direct patient consultation and administrative tasks) was actually significantly higher for patients with NSC than those presenting with a specific complaint. In contrary, material, laboratory and radiology resources were less utilised in patients with NSC. The reasons for this unexpected result are unclear. The clinical reasoning process mainly takes place during the consultation and administrative work, however, from the time spent we cannot deduce anything about the quality of the clinical reasoning process. We assume that physicians seem to sense that these patients with NSC present a challenge, but they just do not seem to know what to do about it. One may further speculate that the non-specific character of these complaints might lead to longer physician-patient consultation and the need for prolonged administrative activities (e.g. contact with primary care physician, collection of past medical history), but may subsequently confuse or even discourage physicians from initiating further diagnostic evaluations. Another possible explanation for the lower utilisation of diagnostic resources might be that attending physicians do not realise the importance of this specific patient group. However, patients hospitalised with NSC tend to suffer from more serious outcomes, as in-hospital mortality and length of stay, thus utilising more resources and generating greater costs [6, 7, 11, 14].

Emergency physicians should be educated about the importance of NSC, in order to prevent underestimation of NSC and a delay in important diagnostic tests. In addition, it remains to be elucidated whether and which subset of patients presenting with NSC might benefit from additional resources spent in the ED to prevent deleterious outcomes. On the other hand, it is not yet clear what kind of resources (i.e. diagnostic studies versus improved clinical reasoning) are needed to have a positive impact. Moreover, cost-effectiveness regarding resource utilisation in the emergency setting versus the admission greatly depends on local settings and warrants further investigations.

In any case, further research is needed to better understand the different patient groups with NSC, as they seem not to be a single homogenous population but a symptom complex including high and low-risk patients.

### Limitations

Our results must be discussed in the light of some limitations. Firstly, this study was conducted at a single centre, albeit one of the largest of its kind in Switzerland, and thus generalisations should only be drawn carefully. However, 69% of the patients were walk-in patients, thus, the selection bias caused by the university character of our hospital might be small. The data used to describe our study population are definitely helpful in comparing another centre to our patient collective and to decide on the transferability of our results. Furthermore, we have developed a resource consumption score of our patient data elsewhere [22]. The identified predictors are widely accepted and even the magnitude of the confounders could be transferred to other hospitals, e.g. Lebanon, so that we are confident that our results are generalisable if handled carefully. Secondly, there is no gold standard definition of NSC. We used the definition introduced by Nemec et al. [13], which we recently demonstrated to be reliable and reproducible, even in a retrospective analysis [11, 30], and that excludes trauma patients. Furthermore, we focussed on patients with highlyurgent or urgent acuteness. Finally, we concentrated on the total diagnostic resources. Future analysis might consider specific steps of the diagnostic work-up of NSC patients as well as the clinical reasoning process [31, 32]. As with all retrospective data analyses, there are some potential layers of bias to be considered [33]. Most importantly, the quality of source data - the medical report and the correct procedural coding - might not be ensured. However, medical reports are usually checked by senior consultants and the staff is regularly trained by controllers to ensure correct procedural coding. Thus, this bias is thought to be small and in addition, can be assumed to be independent of the type of complaint. As regards data abstraction, the abstractors were experienced physicians and the interrater agreement with regard to complaint specificity was very good. In case of discrepancies between the two reviewers, a third reviewer (TCS) was involved.

## Conclusions

Non-specific complaints (NSC) are a frequent reason for emergency medicine consultations and are associated with lower utilisation of diagnostic resources during ED work-up compared to specific complaints.

## Supplementary Information

**Additional file 1: Table S1.** Validation of the diagnosis and drug parser based on agreement with 500 manually coded ED reports.

**Additional file 2: Table S2.** Associations with NSC status.

## Data Availability

Data contain potentially identifying or sensitive patient information. Data used in this study are available from the Emergency Department of the Bern University Hospital, Switzerland upon reasonable request (notfallzentrum@insel.ch) to researchers eligible under Swiss legislation to work with codified personal health care data. Eligibility will be determined by the Bern Cantonal Ethics Committee.

## Abbreviations

CI: Confidence interval
ED: Emergency department
GMR: Geometrical mean ratio
ICU: Intensive care unit
IQR: Interquartile range
LOS: Length of stay
NSC: Non-specific complaints
OR: Odds ratio
SD: Standard deviation

## References

[1] Safwenberg U, Terent A, Lind L (2007). The emergency department presenting complaint as predictor of in-hospital fatality. Eur J Emerg Med.

[2] Bhalla MC, Wilber ST, Stiffler KA, Ondrejka JE, Gerson LW (2014). Weakness and fatigue in older ED patients in the United States. Am J Emerg Med.

[3] Christensen EF, Larsen TM, Jensen FB, Bendtsen MD, Hansen PA, Johnsen SP, Christiansen CF (2016). Diagnosis and mortality in prehospital emergency patients transported to hospital: a population-based and registry-based cohort study. BMJ Open.

[4] Kellett J, Nickel CH (2018). What are nonspecific complaints and what are their causes and outcomes? The common unknown unknowns of medicine. Eur J Intern Med.

[5] Karakoumis J, Nickel CH, Kirsch M, Rohacek M, Geigy N, Muller B (2015). Emergency presentations with nonspecific complaints-the burden of morbidity and the spectrum of underlying disease: nonspecific complaints and underlying disease. Medicine (Baltimore).

[6] Djarv T, Castren M, Martenson L, Kurland L (2015). Decreased general condition in the emergency department: high in-hospital mortality and a broad range of discharge diagnoses. Eur J Emerg Med.

[7] Wachelder JJH, Stassen PM, Hubens L, Brouns SHA, Lambooij SLE, Dieleman JP (2017). Elderly emergency patients presenting with non-specific complaints: characteristics and outcomes. PLoS One.

[8] Quinn K, Herman M, Lin D, Supapol W, Worster A (2015). Common diagnoses and outcomes in elderly patients who present to the emergency department with non-specific complaints. CJEM..

[9] Mockel M, Searle J, Muller R, Slagman A, Storchmann H, Oestereich P, Wyrwich W, Ale-Abaei A, Vollert JO, Koch M, Somasundaram R (2013). Chief complaints in medical emergencies: do they relate to underlying disease and outcome? The Charite Emergency Medicine Study (CHARITEM). Eur J Emerg Med.

[10] Peng A, Rohacek M, Ackermann S, Ilsemann-Karakoumis J, Ghanim L, Messmer AS (2015). The proportion of correct diagnoses is low in emergency patients with nonspecific complaints presenting to the emergency department. Swiss Med Wkly.

[11] Sauter TC, Capaldo G, Hoffmann M, Birrenbach T, Hautz SC, Kammer JE (2018). Non-specific complaints at emergency department presentation result in unclear diagnoses and lengthened hospitalization: a prospective observational study. Scand J Trauma Resusc Emerg Med.

[12] Bingisser R, Dietrich M, Nieves Ortega R, Malinovska A, Bosia T, Nickel CH (2017). Systematically assessed symptoms as outcome predictors in emergency patients. Eur J Intern Med.

[13] Nemec M, Koller MT, Nickel CH, Maile S, Winterhalder C, Karrer C, Laifer G, Bingisser R (2010). Patients presenting to the emergency department with non-specific complaints: the Basel Non-specific Complaints (BANC) study. Acad Emerg Med.

[14] Kemp K, Mertanen R, Laaperi M, Niemi-Murola L, Lehtonen L, Castren M (2020). Nonspecific complaints in the emergency department - a systematic review. Scand J Trauma Resusc Emerg Med.

[15] Safwenberg U, Terent A, Lind L (2008). Differences in long-term mortality for different emergency department presenting complaints. Acad Emerg Med.

[16] Marcozzi D, Carr B, Liferidge A, Baehr N, Browne B (2018). Trends in the contribution of emergency departments to the provision of hospital-associated health care in the USA. Int J Health Serv.

[17] FitzGerald G, Toloo S, Rego J, Ting J, Aitken P, Tippett V (2012). Demand for public hospital emergency department services in Australia: 2000-2001 to 2009-2010. Emerg Med Australas.

[18] Diserens L, Egli L, Fustinoni S, Santos-Eggimann B, Staeger P, Hugli O (2015). Emergency department visits for non-life-threatening conditions: evolution over 13 years in a Swiss urban teaching hospital. Swiss Med Wkly.

[19] Braun CT, Gnagi CR, Klukowska-Rotzler J, Ahmad SS, Ricklin ME, Exadaktylos AK (2017). Trends and weekly cycles in a large Swiss emergency centre: a 10 year period at the University Hospital of Bern. Int J Environ Res Public Health.

[20] Latham LP, Ackroyd-Stolarz S (2014). Emergency department utilization by older adults: a descriptive study. Can Geriatr J.

[21] Hocker MB, Gerardo CJ, Theiling BJ, Villani J, Donohoe R, Sandesara H, Limkakeng AT Jr (2018). NHAMCS validation of emergency severity index as an Indicator of emergency department resource utilization. West J Emerg Med.

[22] Muller M, Schechter CB, Hautz WE, Sauter TC, Exadaktylos AK, Stock S (2021). The development and validation of a resource consumption score of an emergency department consultation. PLoS One.

[23] Exadaktylos AHW (2015). Emergency medicine in Switzerland. ICU Manag.

[24] Rutschmann OT, Hugli OW, Marti C, Grosgurin O, Geissbuhler A, Kossovsky M, Simon J, Sarasin FP (2018). Reliability of the revised Swiss emergency triage scale: a computer simulation study. Eur J Emerg Med.

[25] Methodology WCCfDS. ATC/DDD index. 2018. Available from: https://www.whocc.no/atc_ddd_index/. Accessed 1 Feb 2019.

[26] Gwet KL (2008). Computing inter-rater reliability and its variance in the presence of high agreement. Br J Math Stat Psychol.

[27] Wongpakaran N, Wongpakaran T, Wedding D, Gwet KL (2013). A comparison of Cohen’s Kappa and Gwet’s AC1 when calculating inter-rater reliability coefficients: a study conducted with personality disorder samples. BMC Med Res Methodol.

[28] Maldonado G, Greenland S (1993). Simulation study of confounder-selection strategies. Am J Epidemiol.

[29] Mickey RM, Greenland S (1989). The impact of confounder selection criteria on effect estimation. Am J Epidemiol.

[30] Bingisser R, Nickel CH (2018). Comment to: non-specific complaints at emergencydepartment presentation result in uncleardiagnoses and lengthened hospitalization: a prospective observational study. Scand J Trauma Resusc Emerg Med.

[31] Hautz WE (2018). When I say... diagnostic error. Med Educ.

[32] Zwaan L, Hautz WE (2019). Bridging the gap between uncertainty, confidence and diagnostic accuracy: calibration is key. BMJ Qual Saf.

[33] Kaji AH, Schriger D, Green S (2014). Looking through the retrospectoscope: reducing bias in emergency medicine chart review studies. Ann Emerg Med.

