# Supplementary material for: A dangerously underrated entity? Non-specific complaints at emergency department presentation are associated with utilisation of less diagnostic resources
Source: BMC Emerg Med. 2021 Nov 10;21:133. doi: 10.1186/s12873-021-00531-2 (PMC8582121; doi:10.1186/s12873-021-00531-2)
Supplement: Supplementary file 1 — Additional file 1: Table S1. Validation of the diagnosis and drug parser based on agreement with 500 manually coded ED reports. [file 12873_2021_531_MOESM1_ESM.docx]

**S1 Table. Validation of the diagnosis and drug parser based on agreement with 500 manually coded ED reports.**

|  | **COPD** | **Diabetes** | **Liver disease** | **Dementia** |
| --- | --- | --- | --- | --- |
| **Percent Agreement** | 0.998 (95% CI: 0.994 - 1) | 1 (95% CI: 1 - 1) | 0.998 (95% CI: 0.994 - 1) | 0.996 (95% CI: 0.99 - 1) |
| **Brennan and Prediger** | 0.996 (95% CI: 0.988 - 1) | 1 (95% CI: 1 - 1) | 0.996 (95% CI: 0.988 - 1) | 0.992 (95% CI: 0.981 - 1) |
| **Cohen/Conger's Kappa** | 0.959 (95% CI: 0.878 - 1) | 1 (95% CI: 1 - 1) | 0.97 (95% CI: 0.912 - 1) | 0.748 (95% CI: 0.41 - 1) |
| **Scott/Fleiss' Pi** | 0.959 (95% CI: 0.878 - 1) | 1 (95% CI: 1 - 1) | 0.97 (95% CI: 0.912 - 1) | 0.748 (95% CI: 0.409 - 1) |
| **Gwet's AC** | 0.998 (95% CI: 0.994 - 1) | 1 (95% CI: 1 - 1) | 0.998 (95% CI: 0.994 - 1) | 0.996 (95% CI: 0.99 - 1) |
| **Krippendorff's Alpha** | 0.959 (95% CI: 0.879 - 1) | 1 (95% CI: 1 - 1) | 0.97 (95% CI: 0.912 - 1) | 0.748 (95% CI: 0.409 - 1) |
|  | **Past myocardial infarction** | **Peripheral artery disease** | **Cerebrovascular disease** | **Malignancy** |
| **Percent Agreement** | 0.996 (95% CI: 0.99 - 1) | 0.998 (95% CI: 0.994 - 1) | 0.996 (95% CI: 0.99 - 1) | 0.996 (95% CI: 0.99 - 1) |
| **Brennan and Prediger** | 0.992 (95% CI: 0.981 - 1) | 0.996 (95% CI: 0.988 - 1) | 0.992 (95% CI: 0.981 - 1) | 0.992 (95% CI: 0.981 - 1) |
| **Cohen/Conger's Kappa** | 0.965 (95% CI: 0.915 - 1) | 0.951 (95% CI: 0.856 - 1) | 0.975 (95% CI: 0.941 - 1) | 0.975 (95% CI: 0.941 - 1) |
| **Scott/Fleiss' Pi** | 0.965 (95% CI: 0.915 - 1) | 0.951 (95% CI: 0.856 - 1) | 0.975 (95% CI: 0.941 - 1) | 0.975 (95% CI: 0.941 - 1) |
| **Gwet's AC** | 0.996 (95% CI: 0.989 - 1) | 0.998 (95% CI: 0.994 - 1) | 0.995 (95% CI: 0.989 - 1) | 0.995 (95% CI: 0.989 - 1) |
| **Krippendorff's Alpha** | 0.965 (95% CI: 0.915 - 1) | 0.951 (95% CI: 0.856 - 1) | 0.975 (95% CI: 0.941 - 1) | 0.975 (95% CI: 0.941 - 1) |
|  | **Chronic kidney disease** | **On any antidiabetic (A10)** | **On any antithrombotic (BO1)** | **On any antihypertensive (C02, C04-C09)** |
| **Percent Agreement** | 0.998 (95% CI: 0.994 - 1) | 1 (95% CI: 1 - 1) | 0.99 (95% CI: 0.981 - 0.999) | 0.996 (95% CI: 0.99 - 1) |
| **Brennan and Prediger** | 0.996 (95% CI: 0.988 - 1) | 1 (95% CI: 1 - 1) | 0.98 (95% CI: 0.963 - 0.998) | 0.992 (95% CI: 0.981 - 1) |
| **Cohen/Conger's Kappa** | 0.922 (95% CI: 0.769 - 1) | 1 (95% CI: 1 - 1) | 0.96 (95% CI: 0.925 - 0.995) | 0.985 (95% CI: 0.964 - 1) |
| **Scott/Fleiss' Pi** | 0.922 (95% CI: 0.769 - 1) | 1 (95% CI: 1 - 1) | 0.96 (95% CI: 0.925 - 0.995) | 0.985 (95% CI: 0.964 - 1) |
| **Gwet's AC** | 0.998 (95% CI: 0.994 - 1) | 1 (95% CI: 1 - 1) | 0.987 (95% CI: 0.975 - 0.998) | 0.995 (95% CI: 0.987 - 1) |
| **Krippendorff's Alpha** | 0.922 (95% CI: 0.77 - 1) | 1 (95% CI: 1 - 1) | 0.96 (95% CI: 0.925 - 0.995) | 0.985 (95% CI: 0.964 - 1) |
|  | **On any diuretic (C03)** | **On any opioids (N02A)** | **On any antiepileptic (N03)** | **On any psycholeptic (N05)** |
| **Percent Agreement** | 1 (95% CI: 1 - 1) | 0.996 (95% CI: 0.99 - 1) | 1 (95% CI: 1 - 1) | 0.996 (95% CI: 0.99 - 1) |
| **Brennan and Prediger** | 1 (95% CI: 1 - 1) | 0.992 (95% CI: 0.981 - 1) | 1 (95% CI: 1 - 1) | 0.992 (95% CI: 0.981 - 1) |
| **Cohen/Conger's Kappa** | 1 (95% CI: 1 - 1) | 0.968 (95% CI: 0.925 - 1) | 1 (95% CI: 1 - 1) | 0.977 (95% CI: 0.945 - 1) |
| **Scott/Fleiss' Pi** | 1 (95% CI: 1 - 1) | 0.968 (95% CI: 0.925 - 1) | 1 (95% CI: 1 - 1) | 0.977 (95% CI: 0.945 - 1) |
| **Gwet's AC** | 1 (95% CI: 1 - 1) | 0.995 (95% CI: 0.989 - 1) | 1 (95% CI: 1 - 1) | 0.995 (95% CI: 0.988 - 1) |
| **Krippendorff's Alpha** | 1 (95% CI: 1 - 1) | 0.969 (95% CI: 0.925 - 1) | 1 (95% CI: 1 - 1) | 0.977 (95% CI: 0.945 - 1) |
