# Supplementary material for: A dangerously underrated entity? Non-specific complaints at emergency department presentation are associated with utilisation of less diagnostic resources
Source: BMC Emerg Med. 2021 Nov 10;21:133. doi: 10.1186/s12873-021-00531-2 (PMC8582121; doi:10.1186/s12873-021-00531-2)
Supplement: Supplementary file 2 — Additional file 2: Table S2. Associations with NSC status. [file 12873_2021_531_MOESM2_ESM.docx]

**S2 Table. Associations with NSC status.**

|  | **Specific (n=1310)** | | | **NSC (n=190)** | | **Total (n=1500)** | | | **P-value** |
| --- | --- | --- | --- | --- | --- | --- | --- | --- | --- |
| **Sociodemographic characteristics** |  |  |  | |  | |  |  |  |
| Age, [med (iqr)] | 50.0 | (33-67) | 55.5 | | (39-68) | | 51.0 | (34-67) | 0.027 |
| Sex, [n (%)] |  |  |  | |  | |  |  |  |
| Female | 640 | (48.9) | 73 | | (38.4) | | 713 | (47.5) |  |
| Male | 670 | (51.1) | 117 | | (61.6) | | 787 | (52.5) | 0.007 |
| Swiss nationality, [n (%)] | 927 | (70.8) | 142 | | (74.7) | | 1069 | (71.3) | 0.258 |
| Private insurance, [n (%)] | 171 | (13.1) | 40 | | (21.1) | | 1069 | (71.3) | 0.003 |
| **Time and day** |  |  |  | |  | |  |  |  |
| Saturday or Sunday admission,  [n (%)] | 431 | (32.9) | 51 | | (26.8) | | 482 | (32.1) | 0.095 |
| Night-time admissions, [n (%)] | 593 | (45.3) | 74 | | (38.9) | | 667 | (44.5) | 0.101 |
| Effective weekends, [n (%)] | 519 | (39.6) | 57 | | (30.0) | | 576 | (38.4) | 0.011 |
| Effective days off (weekends  and all public holidays), [n (%)] | 443 | (33.8) | 53 | | (27.9) | | 496 | (33.1) | 0.105 |
| **Comorbidity** |  |  |  | |  | |  |  |  |
| COPD, [n (%)] | 75 | (5.7) | 9 | | (4.7) | | 84 | (5.6) | 0.580 |
| Diabetes, [n (%)] | 153 | (11.7) | 38 | | (20.0) | | 191 | (12.7) | 0.001 |
| Liver disease, [n (%)] | 89 | (6.8) | 16 | | (8.4) | | 105 | (7.0) | 0.411 |
| Dementia, [n (%)] | 27 | (2.1) | 6 | | (3.2) | | 33 | (2.2) | 0.335 |
| Past myocardial infarction,  [n (%)] | 181 | (13.8) | 20 | | (10.5) | | 201 | (13.4) | 0.213 |
| Malignancy, [n (%)] | 204 | (15.6) | 35 | | (18.4) | | 239 | (15.9) | 0.316 |
| Peripheral artery disease, [n (%)] | 42 | (3.2) | 4 | | (2.1) | | 46 | (3.1) | 0.411 |
| Chronic kidney disease, [n (%)] | 51 | (3.9) | 9 | | (4.7) | | 60 | (4.0) | 0.579 |
| Cerebrovascular disease, [n (%)] | 98 | (7.5) | 16 | | (8.4) | | 114 | (7.6) | 0.648 |
| **Drug intake** |  |  |  | |  | |  |  |  |
| On any antidiabetic, [n (%)] | 104 | (7.9) | 27 | | (14.2) | | 131 | (8.7) | 0.004 |
| On any antithrombotic, [n (%)] | 372 | (28.4) | 59 | | (31.1) | | 431 | (28.7) | 0.450 |
| On any antihypertensive, [n (%)] | 411 | (31.4) | 69 | | (36.3) | | 480 | (32.0) | 0.172 |
| On any diuretic, [n (%)] | 178 | (13.6) | 25 | | (13.2) | | 203 | (13.5) | 0.871 |
| On any antibiotic, [n (%)] | 188 | (14.4) | 15 | | (7.9) | | 203 | (13.5) | 0.015 |
| On any antiepileptic, [n (%)] | 108 | (8.2) | 14 | | (7.4) | | 122 | (8.1) | 0.680 |
| On any opioids, [n (%)] | 97 | (7.4) | 15 | | (7.9) | | 112 | (7.5) | 0.810 |
| On any psycholeptic, [n (%)] | 178 | (13.6) | 26 | | (13.7) | | 204 | (13.6) | 0.971 |
| **Consultation characteristic** |  |  |  | |  | |  |  |  |
| Triage category, [med (iqr)] | 3.0 | (2-3) | 3.0 | | (2-3) | | 3.0 | (2-3) | <0.001 |
| High urgent | 588 | (44.9) | 55 | | (28.9) | | 643 | (42.9) |  |
| Urgent | 722 | (55.1) | 135 | | (71.1) | | 857 | (57.1) | <0.001 |
| Resuscitation bay, [n (%)] | 17 | (1.3) | 2 | | (1.1) | | 19 | (1.3) | 0.778 |
| **Outcomes** |  |  |  | |  | |  |  |  |
| Hospitalisation, [n (%)] | 516 | (39.4) | 72 | | (37.9) | | 588 | (39.2) | 0.693 |
| 30 d revisits, [n (%)] | 175 | (13.4) | 22 | | (11.6) | | 197 | (13.1) | 0.497 |
| 365 d revisits, [n (%)] | 476 | (36.3) | 73 | | (38.4) | | 549 | (36.6) | 0.577 |
| LOS hospital days, [med (iqr)] | 0.3 | (0.2-3.4) | 0.3 | | (0.2-3.3) | | 0.3 | (0.2-3.4) | 0.940 |
| LOS ED hours, [med (iqr)] | 4.7 | (3.4-6.5) | 4.9 | | (3.3-6.8) | | 4.7 | (3.3-6.6) | 0.871 |
| ICU admission, [n (%)] | 108 | (8.2) | 13 | | (6.8) | | 121 | (8.1) | 0.507 |
| In-hospital death, [n (%)] | 21 | (1.6) | 5 | | (2.6) | | 26 | (1.7) | 0.310 |
